# Fossil Mice and Rats Show Isotopic Evidence of Niche Partitioning and Change in Dental Ecomorphology Related to Dietary Shift in Late Miocene of Pakistan

**DOI:** 10.1371/journal.pone.0069308

**Published:** 2013-08-02

**Authors:** Yuri Kimura, Louis L. Jacobs, Thure E. Cerling, Kevin T. Uno, Kurt M. Ferguson, Lawrence J. Flynn, Rajeev Patnaik

**Affiliations:** 1 Roy M. Huffington Department of Earth Sciences, Southern Methodist University, Dallas, Texas, United States of America; 2 Department of Geology and Geophysics, University of Utah, Salt Lake City, Utah, United States of America; 3 Peabody Museum and Department of Human Evolutionary Biology, Harvard University, Cambridge, Massachusetts, United States of America; 4 Centre of Advanced Study in Geology, Panjab University, Chandigarh, India; Team ‘Evo-Devo of Vertebrate Dentition’, France

## Abstract

Stable carbon isotope analysis in tooth enamel is a well-established approach to infer C_3_ and C_4_ dietary composition in fossil mammals. The bulk of past work has been conducted on large herbivorous mammals. One important finding is that their dietary habits of fossil large mammals track the late Miocene ecological shift from C_3_ forest and woodland to C_4_ savannah. However, few studies on carbon isotopes of fossil small mammals exist due to limitations imposed by the size of rodent teeth, and the isotopic ecological and dietary behaviors of small mammals to climate change remain unknown. Here we evaluate the impact of ecological change on small mammals by fine-scale comparisons of carbon isotope ratios (δ^13^C) with dental morphology of murine rodents, spanning 13.8 to ∼2.0 Ma, across the C_3_ to C_4_ vegetation shift in the Miocene Siwalik sequence of Pakistan. We applied in-situ laser ablation GC-IRMS to lower first molars and measured two grazing indices on upper first molars. Murine rodents yield a distinct, but related, record of past ecological conditions from large herbivorous mammals, reflecting available foods in their much smaller home ranges. In general, larger murine species show more positive δ^13^C values and have higher grazing indices than smaller species inhabiting the same area at any given age. Two clades of murine rodents experienced different rates of morphological change. In the faster-evolving clade, the timing and trend of morphological innovations are closely tied to consumption of C_4_ diet during the vegetation shift. This study provides quantitative evidence of linkages among diet, niche partitioning, and dental morphology at a more detailed level than previously possible.

## Introduction

Stable carbon isotope ratios (δ^13^C) in bioapatite reflect carbon isotope compositions of diets through physiological processes in animals [1], [2] and have been widely used to reconstruct dietary preferences between C_3_ and C_4_ plants, resource partitioning among coexisting species, and vegetation types in their habitats ([3], [4] and references therein). Stable carbon isotope analysis along with tooth crown height can characterize feeding strategies of fossil mammals [5], [6], [7]. Grimes et al. [8] addressed advantages of using small mammals (e.g., rodents) over large mammals for isotopic paleoenvironmental reconstruction. Higher temporal and spatial resolution would be possible owing to a more abundant fossil record. Stable isotope ratios in small mammals would reflect more local vegetation and environment in their habitats because of their small home ranges without seasonal migration. However, stable isotope studies of small mammals have lagged behind those of large herbivores because obtaining an adequate amount of CO_2_ sample from bioapatite in tiny teeth of small mammals is more difficult. Thus, few studies have been conducted using carbon isotope analysis of rodent teeth. In the previous studies, incisor enamel was preferably used [9], [10], [11], [12] unless molars were large enough to provide the necessary yield of CO_2_ sample from a single individual [13], [14], [15] or molar enamel derived from multiple individuals was combined [16]. Fossil rodents are usually preserved as isolated teeth in sedimentary deposits and are identified almost exclusively based on molar morphology. Isolated incisors themselves can only be classified more-or-less at the order or family level. In using rodent incisors, paleoecological studies of rodents can be done only in those special cases in which incisors are identifiable, for instance, because they are recovered with complete skulls and jaws from a fossil locality with a limited diversity of rodents.

The in-situ infrared laser ablation method improved by Passey and Cerling [17] makes it possible to measure δ^13^C in teeth with 10 to 30 nmol of CO_2_ sample [17], meaning that rodent molars, which are ∼1.5 mm or larger, can generate a single data point from a single specimen using this method. Importantly, post-ablation teeth retain dental morphology for paleontological studies. Hynek et al. [11] applied the laser ablation method to incisor enamel and concluded that rodents are sensitive recorders of paleovegetation by comparing temporal change of δ^13^C in rodents with those of notoungulates and soil carbonates. Gehler et al. [9] suggested that carbon isotope compositions in incisor enamel record dietary differences between modern murine and arvicoline rodents. Therefore, carbon isotope analysis of rodent molars has potential to reveal past ecosystems at more detailed levels than has been achieved with rodent incisors or with large mammals.

Here, we show the significance of using molars of fossil rodents in geochemical analysis along with paleontological approaches to understand interactions between ecology and evolutionary biology of animals by comparing δ^13^C data in molar enamel of murine rodents with morphometric distances in molars that are ecomorphological characters for grazing on finest scale yet achieved through the Miocene Siwalik sequence, Pakistan. The ecological shift from C_3_ trees and shrubs to vegetation dominated by C_4_ grasses was first recognized in Siwalik Group rocks in Pakistan through a temporal sequence of carbon isotopes in soil carbonates [18]. This global event is observed between 8.5 and 6.0 million years (Ma) ago at low to mid-latitudes [19], [20], [21], [22]. The vegetation shift influenced dietary habits of large herbivorous mammals by changing the proportion of plant species available for food ([23] and references therein). Murine rodents from the Siwalik Group were chosen for this study because well-documented δ^13^C data of large herbivorous mammals and soil carbonates from the region are available for comparisons, making the Siwaliks an ideal experimental test, and no other small mammal taxa from the region approach the abundance of murine rodents [24] during the time interval surrounding the ecological and vegetation shift.

Murine rodents (Old World rats and mice) are the most diverse and abundant of modern mammal subfamilies, comprising over 550 species [25]. Siwalik murine fossils represent the best and longest record of murine evolution from the earliest occurrence of the unambiguous murine rodent, *Antemus*, at 13.8 Ma [24]. Their relative abundance increased at an accelerated rate since the first appearance of *Antemus* to become dominant over cricetid rodents at about 11 Ma (YGSP 76), when *Progonomys* appeared [26]. Jacobs [27] and Jacobs and Downs [28] recognized gradual changes of dental morphology through time, which resulted primarily from in-situ evolution in northern Pakistan. They proposed two fundamental lineages derived from *Antemus*: the *Progonomys* clade containing *Progonomys* and *Mus* (mice), and the *Karnimata* clade containing *Karnimata, Parapelomys*, and potentially *Rattus* (rats). The dichotomous lineages are a simplified evolutionary hypothesis but capture overall morphological trends of Siwalik murine rodents [27], [28], [29]. Thus, the effect of ecological change on the evolution of small mammals can be most effectively tested by utilizing the finely-spaced fossil record of Siwalik murine rodents with minimum influence of immigration from other regions.

## Materials and Methods

### Study design

We measured δ^13^C values in lower first molars (m1) of murine fossils collected from the Siwalik Group in the Potwar Plateau of northern Pakistan, ranging from13.8 to 6.5 Ma (21 localities), and northern India, ranging from ∼2.5 to ∼1.8 Ma (2 localities), and compared them with the compiled data of large herbivorous mammals and soil carbonates presented in Figure 2 of Badgley et al. [23]. Carbon isotopes in enamel reflect dietary input during the time of tooth formation, whereas dental morphological characters are long-term adaptive traits obtained through evolutionary history. Two ecomorphological characters, van Dam's [30] index (VD index hereafter, defined below) and tooth crown height (hypsodonty) were evaluated to compare timing and direction of morphological change with isotopic dietary inferences.

To minimize inter-tooth isotopic variation, we deliberately analyzed only m1 (except several samples from the Pliocene of India). Isotope compositions across teeth in different tooth positions are influenced by nursing to different degrees (Figure S1) because mineralization of molar enamel starts before weaning in murine rodents (see Supporting Information S1). Although we do not exactly know the carbon isotope effect of nursing on murine molars, the magnitude of relative change across the data of m1 should reflect actual changes in the Siwalik murines, assuming that physiological differences in the closely-related species are negligible. We chose m1 over the second and third molars even though m1 is most influenced by mother's milk because m1 is large enough (1.5 to 2.9 mm in length) to generate a CO_2_ sample from a single tooth, and morphological complexity of m1 is useful for systematic identification and allows for species-level comparisons.

### Materials

Samples ranging in age from 13.8 to 6.5 Ma were collected by sieving in the 1970's to 2000 from the Potwar Plateau, northern Pakistan. The fossil specimens are on long-term loan from Pakistan, under the authority of the Geological Survey of Pakistan, Islamabad, Pakistan, and are housed in the Peabody Museum of Archaeology and Ethnology, Harvard University. To fill the time gap between 6.5 Ma and Recent, five specimens from Kanthro (∼2.5 Ma) and six specimens from Nadah (∼2.0 to ∼1.8 Ma) recovered from Upper Siwalik localities of northern India [31], [32] were analyzed. All Indian specimens used in this study are housed in the Centre of Advanced Study in Geology, Panjab University. Recent specimens from both regions were also utilized for comparison. Among those from Pakistan, *Golunda ellioti* and two *Mus* species (*M. booduga, M. saxicola*) were from owl pellets collected on the Potwar Pleatau, whereas *Rattus* sp. and *Millardia* sp. were captured in urban areas of the Potwar between 1970 and 1975. They are housed in the Shuler Museum of Paleontology, Southern Methodist University. *Bandicota indica* was collected from an owl pellet in Nagar, Punjab State, India in 2011. Specimen numbers are given in Datasets S1 and S2. No additional permits were required for the described study, which complied with all relevant regulations.

### Specimen Identification and Sample Selection

Jacobs [27], [33] described rodent specimens from localities YGSP 491 (13.8 Ma), YGSP 41 and 430 (13.6 Ma), YGSP 182 (9.2 Ma), DP 13 (6.5 Ma), and DP 24 (∼1.7 Ma), and recognized 9 species among 8 genera. Cheema et al. [34] described *Progonomys hussaini* from locality JAL-101 (∼11 Ma). Specimens from other localities have been identified at the generic level or, if not, were assigned to ambiguous groups such as “near *Antemus*” according to the most updated study by Jacobs and Flynn [29]. The qualitative nature of the taxonomic assignments and anagenetic change in time-consecutive taxa impede the systematic classification of the Siwalik murines. We grouped tooth samples at each stratigraphic level based on size and morphology, so that individuals in the same assemblage display high similarity and generally followed Jacobs and Flynn [29] for names of taxa (Table S1). We analyzed all murine species known with n>5 by upper first molar (M1) at each stratigraphic level, as well as large *Karnimata* sp., which are minor components (n<5 by M1) at 8.2 and 8.8 Ma (Table S1). Complete m1 with slight to moderate wear, corresponding to wear stage I to IV of Lazzari et al. [35], were selected for carbon isotope analysis. The tooth samples are neither weathered nor etched. The enamel is intact based on visual observation under a light microscope with 100× magnification for all and SEM observation for randomly selected specimens. Diagenetic alteration was assumed to be negligible for the murine rodent teeth as confirmed by Quade et al. [36] for large mammal teeth from the Siwalik sediments of similar ages in the same region.

### Carbon Isotope Analysis

We conducted in-situ laser ablation GC-IRMS isotope analyses on enamel of m1 using the laser ablation method described by Passey and Cerling [17] at the University of Utah. Isotope ratios are expressed as δ values (‰) on VPDB scale, where δ_sample_  =  (R_sample_/R_standard_-1)*10^3^, and R is given by ^13^C/^12^C. Fossil specimens were cleaned by acetone to remove glue and were treated with 2% NaOCl for one hour to remove organic contaminants, followed by 0.1 M sodium acetate-acetic acid buffer for one hour (except specimens from YGSP 182, see below) to remove diagenetic carbonate minerals from enamel surfaces. The time period for the 2% NaOCl treatment varied from 3 hours to 8 hours in modern specimens, depending on the amount of organic tissue adhered to the specimen. The 0.1 M acetic acid buffer (pH = ∼5.4) was used rather than 0.1 M acetic acid (pH = ∼3.0) because thin enamel of murine molars are etched by 0.1 M acetic acid in only 15 minutes as observed under SEM (Figure S2). The treated samples were mounted on a plate of the laser sample chamber using Bostik Blu-Tack. The lingual side was preferentially used, but the labial and posterior sides were also analyzed when data results from the lingual side were not reliable due to low CO_2_ yield or contamination of CO_2_ generated from ablation of dentine or Blu-Tack. CO_2_ laser (wave length: 10.6 μm) setting ranged from 1.8 to 7.5 W with a 8.5 ms pulse duration. CO_2_ from multiple ablation pits was cryogenically concentrated, inlet to a gas chromatographic column (Poraplot Q; 60°C) and sent into a Finnigan MAT 252 gas-ratio mass spectrometer via a GC/CP interface.

Isotope data reported in this study were corrected for isotope fractionation between laser and conventional H_3_PO_4_ methods to be comparable with the compiled data of Badgley et al. [23], in which carbon isotope values were obtained by the H_3_PO_4_ method. Isotope enrichment (ε) between two substances A and B is defined as ε_A−B_  =  (R_A_/R_B_ −1)*1000 [2], [37], which is independent on the scale (i.e., PDB, SMOW) and more preferable than the scale-specific difference of Δ_A−B_  =  δ_A_–δ_B_ [2], [38]. The superscript * (ε*) is designated for non-equilibrium processes [2]. The mean ^13^ε*_laser-H3PO4_ value was −1.5±0.3‰ (1σ) for a modern beaver incisor analyzed in every run, whose isotope values had been determined by the H_3_PO_4_ method. This value is larger than the value (^13^ε*_laser-H3PO4_ = −0.3‰) reported by Passey and Cerling [17] but is smaller than those of Podlesak et al. [39]. The mean ^18^ε*_laser-H3PO4_ value, −6.1±0.9‰, was a much larger offset than that of ^13^ε*_laser-H3PO4_ values. This is because laser ablation of enamel liberates both phosphate and carbonate bound oxygen, whereas the H_3_PO_4_ method targets only carbonate oxygen. There is a ∼9 permil offset between phosphate and carbonate bound oxygen [40]. Oxygen isotope data produced by the CO_2_ laser ablation method are less precise than isotopic measurements of phosphate oxygen or structural carbonate oxygen due to incomplete mixing of oxygen-bearing components [17]. We do not discuss oxygen isotope compositions of Siwalik murine rodents in this study. Carbon isotope compositions of Recent species reflect light carbon inputs to the atmosphere by burning fossil fuels. They were corrected for δ^13^C values (−6.5‰) of atmospheric CO_2_ in pre-industrial times [41]. Modern δ^13^C _atmosphere_ in years of sampling (1970's and 2011) are −7.4‰ [42] and −8.2‰ [43], respectively.

Nine specimens from YGSP 182 (Dataset S1) were treated with 0.1 M acetic acid for 8 hours and resulted in strongly etched enamel surface. These initial treatment results led us to check the effect of the acetic acid treatment on murine enamel. By observation under SEM, removal of non-prismatic outer enamel started in only 15 minutes in a 0.1 M solution due to the low pH of the acetic acid (pH = ∼3.0) (Figure S2). Thus, the 0.1 M acetic acid treatment was replaced with 0.1 M sodium acetate-acetic acid buffer (pH = ∼5.4). The isotopic composition of the YGSP 182 specimens may have shifted by the acetic acid treatment. In Koch et al. [44], powdered enamel samples treated with 0.1 M acetic acid for three days were altered by −0.2 ‰ for δ^13^C and by +0.9 ‰ for δ^18^O compared to untreated samples. In the YGSP 182 specimens, the mean isotope ratio of the etched samples (n = 9) are offset by −1.1‰ in carbon and by +1.5‰ in oxygen, compared to samples (n = 2) treated by buffered acetic acid. Although the direction of the acid effect is the same as demonstrated in Koch et al. [44], the absolute values for correction are not known in this study. The nine specimens are not corrected for the difference in the acid treatment.

### Quantifying Dental Characters

The VD index evaluates space between anteroposteriorly aligned cusps on upper first molar (M1), defined as the ratio of tooth width to a distance between the posterior side of the lingual anterocone and that of protocone. Species with a value of more than 2.2 are predominantly grazers [30], [45]. Note that van Dam [30] used two other characters along with VD index in a principle component analysis in order to distinguish grazing murines from non-grazers. Both characters are based on m2, which are more difficult to identify and are not used in this study. Following the result of van Dam [30], taking 2.2 as a critical value for grass diets is reasonable although it does not necessarily mean that every species with >2.2 is a grazer.

Hypsodonty is well-studied in ungulates and is associated with adaptation to high rates of tooth wear due to tough diets together with ingestion of soil and grit while feeding [46], [47]. Hypsodonty index is most commonly determined by height/width of unworn m3 [48]. In this study, hypsodonty was measured as crown height divided by length of M1. The length was used instead of width because its variance was less than half that of width among the species identified by Jacobs [27], [33].

Specimens used for VD index are unworn to moderately worn, corresponding to wear stage I to IV of Lazzari et al. [35], and those used for hypsodonty are unworn or slightly worn specimens in wear stage I to II. All dental measurements were taken in VHX-1000 communication software based on 2D digital pictures photographed with a Keyence VHX-1000 digital microscope. In a mean of VD index for each species, large *Karnimata* sp. at 9.2 Ma (YGSP 7717) was included in *K*. *darwini* due to small sample size.

### Statistical Analysis

The rates of temporal change in δ^13^C values were compared between two anagenetic lineages, *Karnimata* of the *Karnimata* clade (blue circles in figures) and the *Progonomys* clade without *Mus* sp. at 7.4 Ma (red triangles in figures), from 9.2 through 6.5 Ma by using linear regression lines between δ^13^C values (dependent variable) and time (independent variable, covariate) of the two clades (categorical variable). That species of *Mus* sp. was excluded because it may be an immigrant from another region, based on its smaller size and more elongated anterior side of the lingual and labial anterocones than those of the younger *Mus auctor* (6.5 Ma). To avoid confusion, the *Progonomys* clade without *Mu*s sp. at 7.4 Ma is expressed as the “*Progonomys* clade” hereafter. We tested the interaction between the categorical variable and the covariate in the aov() command of R for a null hypothesis that they do not interact. Because the null hypothesis was accepted, i.e., slopes of the two regression lines are equal (*p* = 0.54), Analysis of covariance (ANCOVA) was performed to test if intercepts of the linear regression lines between the two clades are significant. Before performing the linear regression, we checked that a linear model fitted the data as well as or better than a quadratic model. Temporal changes in VD index and hypsodonty values between the two clades were also examined in the same procedure. We did not adopt linear regression analysis between the ecomorphological characters and δ^13^C values (e.g., VD index vs. mean δ^13^C values) because the variables are at different time scales. Carbon isotope compositions in enamel are a short-term variable, reflecting mixing of two end-members, C_3_ plants and C_4_ plants, in their diets during enamel mineralization. On the other hand, VD index and hypsodonty are long-term variables that species acquired along phylogeny. As a result, linear regressions between the ecomorphological characters and δ^13^C values have lower R^2^ values than linear regressions of temporal changes in the three variables.

At 7.4 Ma, 6.5 Ma, and Recent, in which three species were considered, Welch's ANOVA was performed to assess whether means of δ^13^C values in the three coexisting species are indistinguishable at α = 0.05. Welch's ANOVA was chosen to avoid increasing Type II error rates due to small sample sizes. The Games-Howell test was used for post-hoc tests to identify the location of significant differences at α = 0.05.The assumptions of normality and homogeneity of variance were checked by the Shapiro-Wilk test and the Bartlett's test. Recent *Millardia* sp. was combined with Recent *Rattus* sp. due to small sample size. Although molecular studies show they are not very closely related [49], *Millardia* was sometimes considered a subgenus of *Rattus* [50]. Recent *Mus* spp. includes *Mus booduga* and *Mus saxicola*. The 95% bootstrap confidence intervals were computed by the bias-corrected and accelerated method with 9999 randomizations in the ‘boot’ package [51]. All statistical tests but Welch's ANOVA and Games-Howell test, which were computed in IBM SPSS Statistics 18 [52], were performed in R2.15.1 [53]. Samples whose values clearly deviate from others in the same species and are beyond three standard deviations were removed from statistical tests as outliers.

## Results

**Figure 1:**
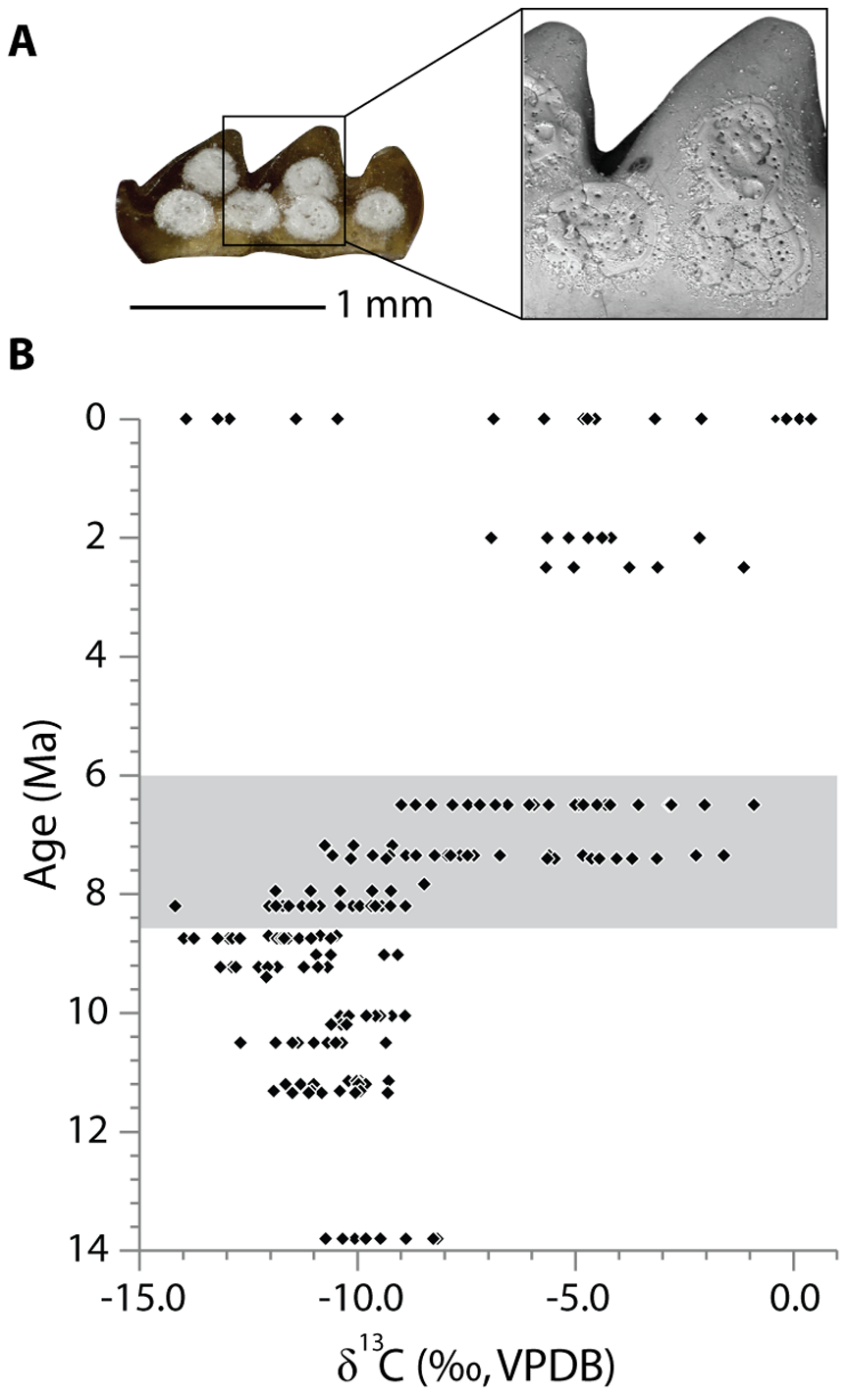
(A) Laser pits on the lingual side of YGSP 34539 (reversed, anterior to left). (B) Carbon isotope ratios of enamel in the lower first molars (m1) of Siwalik fossil and Recent murines. Ages of the Siwalik localities derived from paleomagnetic stratigraphy are based on Geomagnetic Polarity Time Scale of Ogg and Smith [63].

Stable carbon isotope data combined for all murine species are shown in Figure 1, and summarized in Table 1 and Table S2 (Dataset S1). A total of 189 carbon isotope data points were obtained from modern and fossil murines from the Siwalik Group. Each data point is represented by a single molar.

**Table 1:** Carbon isotope data summarized by species. Note that all data are included in the descriptive statistics.

Age (Ma)	Species	N	Mean	SD	Min	Max	Combined locality/age
Recent	*Golunda ellioti*	5	−0.8	2.3	−4.8	0.4	
	*Rattus* sp	3	−11.9	1.8	−13.9	−10.5	
	*Millardia* sp	3	−10.6	4.2	−13.2	−5.7	
	*Mus booduga*	4	−4.2	2.1	−6.9	−2.1	
	*Mus saxicola*	1	−4.7				
	*Bandicota indica*	2	−2.1	0.0	−2.1	−2.1	
∼2.5 to ∼1.8	*Golunda* spp	4	−2.5	1.1	−3.8	−1.1	loc. Kanthro, loc. Nadah
	*Parapelomys robertsi*	1	−5.0				
	*Bandicota* sp	1	−4.7				
	*Cremnomys* spp	4	−5.2	0.6	−5.7	−4.4	
	*Mus cf. M. flynni*	1	−6.9				
6.5	*Parapelomys robertsi*	4	−8.1	1.0	−9.0	−6.8	
	*Karnimata huxleyi*	8	−3.8	1.8	−6.1	−0.9	
	*Mus auctor*	10	−5.6	1.7	−8.3	−2.8	
7.2	*Karnimata* sp	1	−10.1				
	*Mus* sp	2	−10.0	1.1	−10.7	−9.2	
7.4	*Parapelomys* sp	1	−4.4				
	*Karnimata* sp	13	−6.1	2.4	−9.7	−1.6	
	*Progonomys* sp	8	−7.2	2.7	−10.2	−3.1	
	*Mus* sp	3	−7.8	3.4	−10.6	−4.1	
8.2	*large Karnimata* sp	2	−9.4	0.2	−9.6	−9.2	8.0 Ma, 8.2 Ma
	*Karnimata sp*	10	−10.4	1.4	−14.2	−8.9	
	*Progonomys* sp	10	−11.1	0.9	−12.0	−9.4	
8.8	*large Karnimata* sp	3	−11.0	0.4	−11.3	−10.6	8.7 Ma, 8.8 Ma
	*Karnimata* sp	2	−12.4	0.5	−12.7	−12.0	
	*Progonomys* sp	12	−12.3	1.0	−14.0	−10.9	
9.0	*Karnimata darwini*	1	−9.1				
	*Progonomys debruijni*	3	−10.3	0.8	−10.9	−9.4	
9.2	*Karnimata darwini*	10	−12.0	0.8	−13.1	−10.7	9.2 Ma, 9.4 Ma
	*Progonomys debruijni*	2	−12.4	0.5	−12.8	−12.1	
10.1	*Karnimata sp. + Progonomys* sp	10	−9.6	0.5	−10.4	−8.9	
10.5	*Karnimata sp. + Progonomys* sp	13	−10.9	0.8	−12.7	−9.3	10.2 Ma, 10.5 Ma
11.2	*Progonomys hussaini + ? Karnimata* sp	8	−10.4	0.8	−11.6	−9.3	
11.4	*Progonomys hussaini*	10	−10.6	0.8	−11.9	−9.3	11.3 Ma, 11.4 Ma
13.8	*Antemus chinjiensis*	8	−9.5	0.9	−10.7	−8.2	

The δ^13^C values became abruptly more positive with a broader range at 7.4 Ma. The mean δ^13^C value is −10.8‰ for pre-7.4 Ma (i.e., 13.8 to 7.8 Ma) and becomes −6.6‰ at 7.4 Ma. The range value of 9.0‰ at 7.4 Ma is a threefold increase compared to previous age intervals. Predating the rapid increase, there is a gradual negative shift of 2.5‰ between 13.8 Ma and 8.7 Ma. A relatively large offset at 9.2 Ma compared to 9.0 Ma may be partly attributed to the difference in the acid procedure (see Carbon Isotope Analysis in Material and Methods). From 8.7 to 8.2 Ma, the mean δ^13^C value shifted by +1.5‰ without changing the range of variation. Despite a slight increase from 8.7 to 8.2 Ma, the maximum values at 8.2 Ma and 8.0 Ma do not exceed the maximum value at 13.8 Ma.

The rates of temporal change in δ^13^C values are not significantly different (*p* = 0.54, Figure S3, Table S3) between *Karnimata* (large species, blue circles in figures) and the “*Progonomys* clade” (small species, red triangles in figures), indicating both clades experienced the same degree of dietary change from 9.2 through 6.5 Ma. ANCOVA shows intercepts of the regression lines are significantly different between the clades (*p* = 0.002, Table S3), meaning that *Karnimata* species have a greater mean δ^13^C value than the “*Progonomys* clade” when the effect of time is controlled. Each linear model predicts 73% and 69% of the variance in δ^13^C values by time, respectively (*p*<0.001, Table S4). Based on the linear regression lines, *Karnimata* species and the “*Progonomys* clade” increase δ^13^C values by 3.0‰ and 2.8‰ per million years, respectively.

The VD index of *Karnimata* is positively related to time (R^2^ = 0.56, *p*<0.001), whereas there is no significant linear relationships between VD index and time in the “*Progonomys* clade” (Table S4, Dataset S2). The rates of temporal change in VD index are significantly different (*p*<0.001, Figure S4, Table S3) between the clades, indicating that *Karnimata* species increase VD index values through time, while VD index is constant in the “*Progonomys* clade”. Hypsodonty shows no relationship with time in either clade (Table S4, Dataset S2), but ANCOVA shows the observed difference in the intercepts is marginally significant (*p* = 0.04, Figure S5, Table S3), meaning that *Karnimata* species have higher tooth crowns than the “*Progonomys* clade”.

Statistical tests showed significant differences in mean δ^13^C values among coexisting species at 7.4 Ma, 6.5 Ma, and Recent (Tables S5 and S6). For 7.4 Ma and 6.5 Ma, post-hoc tests show a mean of one species is significantly different from those of other coexisting species. At 7.4 Ma, the smallest species, *Mus* sp. (green diamonds in figures), has a mean δ^13^C value more negative than *Progonomys* sp. and *Karnimata* sp. by 2.6‰ and 3.5‰, respectively. At 6.5 Ma, a mean δ^13^C value of the largest species, *Parapelomys robertsi* (light blue squares in figures), is more negative than that of *Mus aucto*r and *Karnimata huxleyi* by 2.4‰ and 4.3‰, respectively.

## Discussion

### Comparisons with Carbon Isotope Ratios in Large Mammals and Soil Carbonates

An abrupt positive shift of δ^13^C values indicates a pronounced dietary shift of murine rodents between 7.8 and 7.4 Ma. The dietary shift is more abrupt in murine rodents (Figure 1) than in large mammals [23]. Comparisons at different systematic levels may contribute to the fact that large mammals have wider variation of δ^13^C values than murine rodents. However, the ranges of δ^13^C values in murine rodents are concordant with variation within 5 to 95 percentiles of δ^13^C values in large mammals, ∼3‰ for 13 to 11 Ma and ∼9‰ for 7.4 to 7.0 Ma. Hipparionine equids incorporated C_4_ grasses into their diet by ∼8.5 Ma [19], [23], whereas murine rodents lagged behind these ungulates by one million years. The pattern of δ^13^C values in murine rodents is similar to the isotopic shift recorded in soil carbonates, which reflect the overlying vegetation more directly than large mammal data. It suggests that murine rodents record past ecological conditions more precisely than large mammals because of their feeding behavior as dietary opportunists with small home ranges in short life span.

In contrast, neither large mammals nor soil carbonates record the gradual negative shift of 2.5‰ toward 8.7 Ma observed in murine data. The negative shift may reflect an actual change in vegetation because the most negative mean δ^13^C values in large mammals and soil carbonates are at 9.2 to 8.6 Ma and at 9.2 to 8.3 Ma, respectively, which encompasses the 8.7 Ma level of the murine data.

### Dietary Niche Partitioning

**Figure 2:**
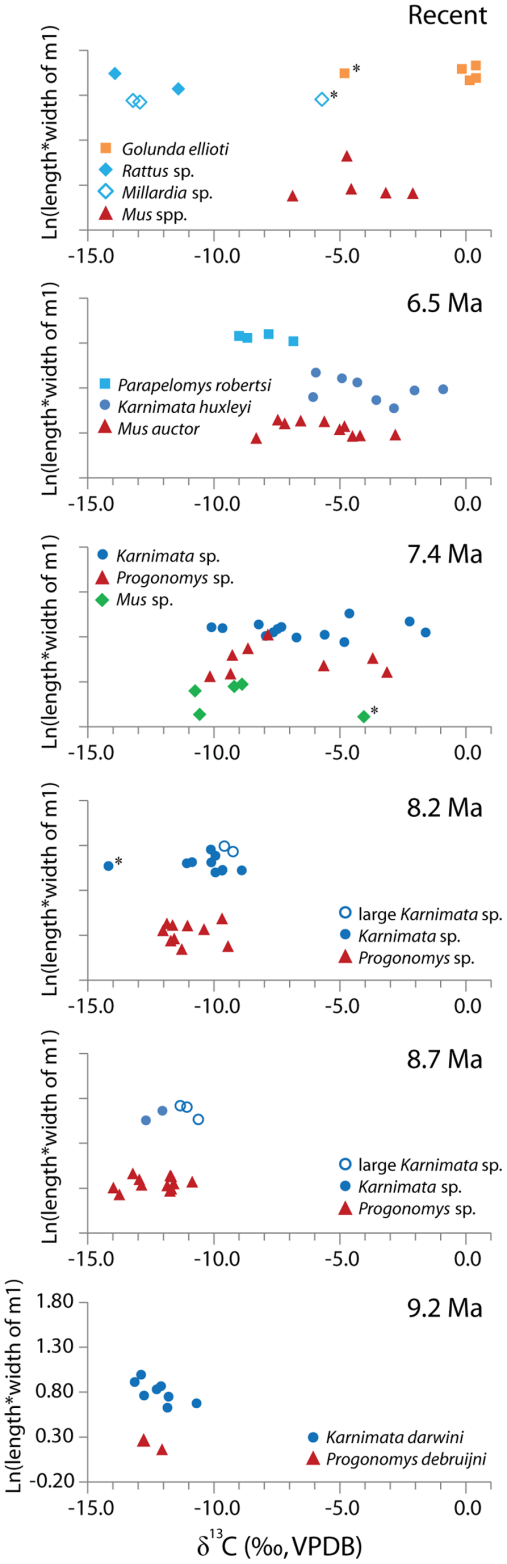
Scatter plots of δ^13^C values vs. the natural logarithm of tooth size, Ln(length*width), of m1 in murine rodents, ranging from 9.2 Ma to 6.5 Ma and Recent. Outliers removed from statistical tests are shown by asterisks.

From 9.2 Ma through 6.5 Ma, large murine species have a consistently greater mean δ^13^C value than small species with the exception of *Parapelomys robertsi* (Figure 2). Carbon isotope ratios in coexisting species can vary due to different patterns in spatial occupation. In a modern ecosystem, Codron et al. [54] showed δ^13^C values of C_3_ plants vary by up to 2‰ in an African savanna, having more negative values associated with availability of perennial water sources. Thus, the isotopic variation (less than 1.7‰) between large and small murine species observed from 9.2 Ma through 8.2 Ma may be explained by spatial partitioning, with small species feeding on less water-stressed C_3_ plants. Considering isotopic enrichment of insects and non-photosynthetic plant tissues relative to green leaves [55], [56], the magnitude of the isotopic variation can also be explained by dietary niche partitioning, with large species feeding on more strictly vegetarian diets. The intake of C_4_ grasses might be partially responsible for the isotopic differentiation as early as 8.2 Ma because isotopes in hipparionine equids indicate a C_4_ dietary component beginning at 8.5 Ma in Pakistan [23]. Nevertheless, C_3_ vegetation still dominated major floodplains at ∼8.0 Ma [57].

The isotopic variation of less than 2‰ may also result from proportional contributions of insects and non-photosynthetic tissues of C_3_ plants in the diet of murine rodents. Most of today's murine rodents are omnivorous [50], consuming a variety of plant material (seeds, grains, nuts, fruits, leaves, stems, roots), insects, and other invertebrates. Insects tend to be more enriched in ^13^C than primary producers due to trophic enrichment [55]. The mean estimate of trophic shift is +0.6‰ among terrestrial arthropods, which are one of major food source for murines, based on data compiled in McCutchan et al. [55]. The isotopic differentiation resulting from different proportional amounts of insects is observed in modern *Rattus rattus* and *Mus musculus* inhabiting forests of Hawaii, where they partition food resources in that *Rattus rattus* is primarily vegetarian (∼80% of stomach content being fruits and seeds), whereas *Mus musculus* consumes a large amount of arthropods (∼50% arthropods, ∼30% fruits and seeds) [58]. A mean δ^13^C value in bone collagen of *Rattus rattus* is more negative than the more insectivorous *Mus musculus* by ∼1.4‰ [58]. Non-photosynthetic plant tissues are slightly more enriched in ^13^C than leaves [59]. Badeck et al. [56] documented that roots and woody stems are isotopically heavier than leaves by 1.1‰ and 1.9‰ on average in C_3_ plants, respectively, whereas the differences between roots and leaves are not significant in C_4_ plants. Fruits and seeds are also more enriched than leaves. The isotopic differences between fruits and leaves (Δ^13^C _fruits-leaves_) range from 0.5‰ to 3.0‰ in various species of leguminous plants [60], [61].

By 7.4 Ma, the difference in mean δ^13^C values between large (*Karnimata* sp.) and small (*Mus* sp.) species increased significantly, reaching 3.5‰ (Figure 2 and Table S6). The difference of 3.5‰ is too great to be explained solely by isotopic variation in C_3_ plants or physiological variation in murine species and therefore indicates that consumption of C_4_ grasses had come to play an important role in isotopic differentiation among coexisting species. If the difference arose solely from proportional contribution of C_4_ to C_3_ diets, we conclude that *Karnimata* sp. preferentially consumed C_4_ plants over C_3_ green leaves 20 to 30% more than *Mus* sp. at 7.4 Ma. The isotopic difference of 4.3‰ at 6.5 Ma, between *Parapelomys robertsi* and *Karnimata huxleyi*, translates into 30 to 40% more consumption of C_4_ grasses by *K. huxleyi*.

### Comparisons with Dental Morphology

**Figure 3:**
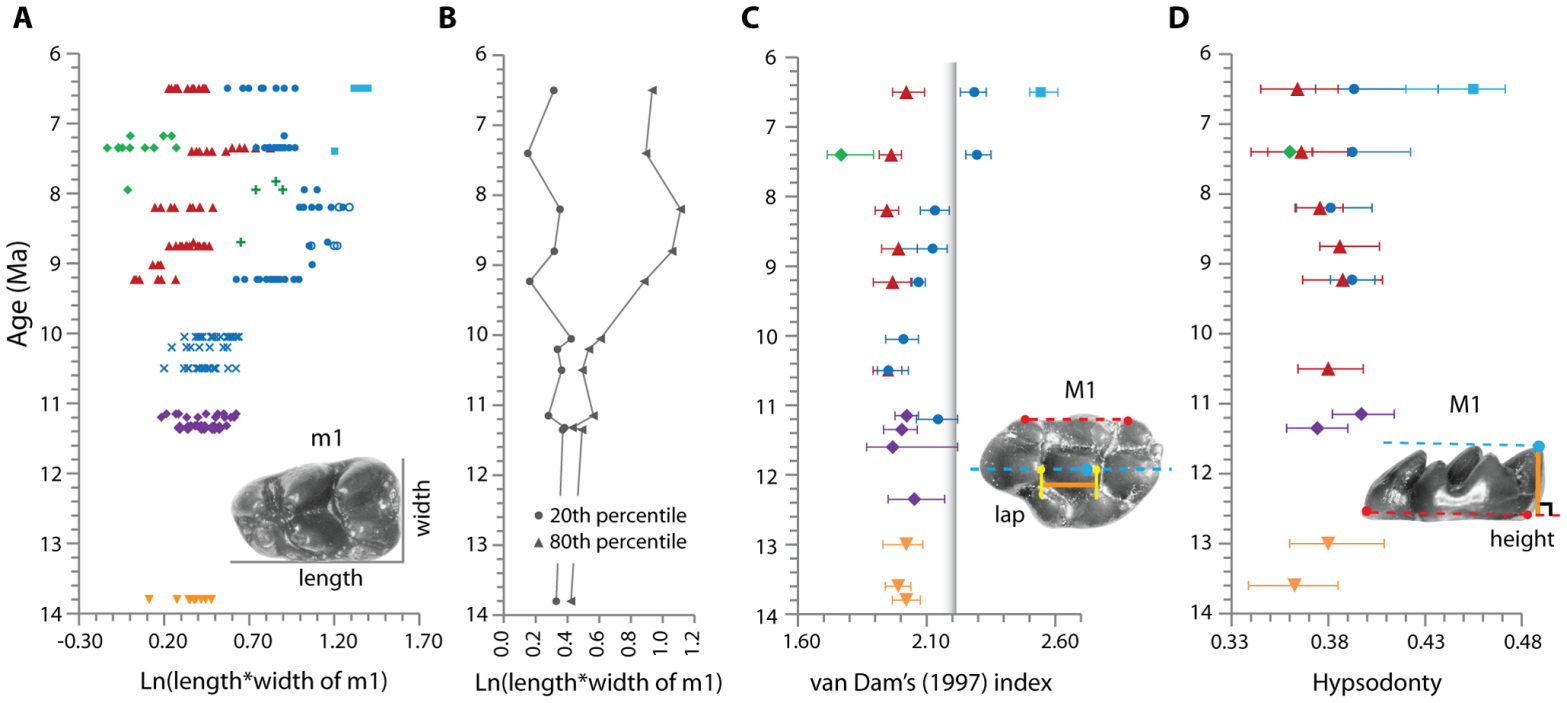
Change in morphological characters through time. (A) Ln(length*width of m1). Symbols in A: 13.8 to 13 Ma, inverted orange triangle for *Antemus chinjiensis*; 11.6 to 11.2 Ma, purple diamond for *Progonomys hussaini* +?*Karnimata* sp.; 10.5 to 10.1 Ma, blue cross for *Karnimata* sp. + *Progonomys* sp.; 8.8 Ma, green cross for morphotype 7; 7.8 Ma, green cross for morphotype 8; 7.4 Ma, light blue square for *Parapelomys* sp.; other symbols as in Figure 2. (B) 20th percentile and 80th percentile of Ln(length*width of m1). (C) van Dam's [30] index of M1, calculated as tooth width divided by the distance (lap in figure) between the posterior side of the lingual anterocone and that of protocone. The vertical bar at 2.2 shows the lower boundary of VD index to be predominantly grazers. Symbols in C: 12.4 Ma, purple diamond for near *Progonomys* sp.; 11.6 to 11.2 Ma, purple diamond for *Progonomys hussaini*; 11.2 Ma, blue circle for ?*Karnimata* sp.; 10.5 and 10.1 Ma, blue circle for *Karnimata* sp., red triangle for *Progonomys* sp.; 8.8 and 8.2 Ma, blue circle for *Karnimata* sp. (+ large *Karnimata* sp.); other symbols as in A. (D) Hypsodonty of M1, calculated as crown height divided by length. Symbols as in C. Error bars indicate 95% bootstrap confidence intervals. Red dotted lines connect two reference points, and blue dotted lines are parallel to the red line and have one reference point.

The tooth size of each individual is expressed as a natural logarithm of tooth area, Ln(length*width), in Figures 2 and 3A, B (Table S7). Two sympatric species (or morphotypes) can be recognized as early as 11.2 Ma based on dental morphology of M1 [29]. These species greatly overlap in size and morphology of m1. They are tightly clustered by carbon isotope composition (Figure 1, Table 1), indicating that these species of similar size did not isotopically partition their diets. This condition lasted at least until 10.1 Ma. Size divergence occurred by 9.2 Ma (Figure 3A, B). Compared to corresponding species at 8.2 Ma, by 7.4 Ma, *Progonomys* sp. became larger, while *Karnimata* sp. reduced its size, resulting in opening morphospace for small *Mus* sp. and large *Parapelomys* sp. The species of *Mus* sp. was a major species at 7.4 Ma (Figure 3A). The presence of three major species (*P. robertsi, K. huxleyi, M. auctor*) continued through 6.5 Ma (Table S1).

Among coexisting species that differ in size, large species consistently have greater VD index and higher hypsodonty values (i.e., narrower valleys between anteroposteriorly aligned cusps on higher-crowned teeth) than small species from 9.2 through 6.5 Ma (Figures 3C, D, Tables S8 and S9). *Karnimata* sp. progressively narrowed valleys between anteroposteriorly aligned cusps through the evolutionary sequence, indicating that they consumed greater amounts of tough diets through time. In the VD index, *Karnimata* sp. exceeds the grazing value of 2.2 by 7.4 Ma, and large species (*Parapelomys robertsi* and *Karnimata huxleyi*) are above that value at 6.5 Ma (Figure 3C). In *Karnimata*, the attainment of the VD value signaling consumption of grass diets corresponds in time to the increase of the mean δ^13^C value and increased total range of δ^13^C values at 7.4 Ma. On the other hand, the “*Progonomys* clade” did not modify the valley space between cusps as shown by a constant VD index value even though the rate of dietary change toward more tough diets is same as *Karnimata*. At 7.4 Ma, *Mus* sp. has a mean VD value (1.77) significantly lower than that of *Progonomys* sp (1.96). Although the rates of change in δ^13^C values are the same in both clades, the different rates of morphological evolution in VD index indicate that selection pressures leading to the morphological change are greater in *Karnimata* than in the “*Progonomys* clade”.

There is no evidence of increasing crown height in *Karnimata* and the “*Progonomys* clade”, but *Parapelomys robertsi*, which is a derived member of the *Karnimata* clade [28], has significantly higher tooth crowns than the coexisting species at 6.5 Ma (*p*≤0.01 for each pair in the Games-Howell test). Thus, in Siwalik murines, tooth crown height lagged attainment of the grazing threshold in the VD index by 2.7 Ma. A morphological trend toward increasing VD index and hypsodonty values was also recognized in a clade of European murine rodents, *Progonomys-Occitanomys-Stephanomys* [30].

### Contradictions in Carbon Isotope and Dental Morphology of P. robertsi

At 6.5 Ma, the largest species, *Parapelomys robertsi*, is more depleted in ^13^C than its two coexisting species (*Mus auctor, Karnimata huxleyi*), which would indicate a higher proportion of C_3_ plants in the diet. However, *P. robertsi* is expected to be a grazer based on dental characters. Its VD index value (2.5) is equivalent to that of Recent *Golunda ellioti* (Table S8), which predominantly consumes C_4_ grass. Its tooth crown is higher than any other Siwalik murines (Figure 3D and Table S9). Similar apparent contradictions in dental morphology and isotopic indicators of diet can be found in modern rodents. Recent *Millardia* sp. shows more negative δ^13^C values than Recent *Mus booduga* and *Mus saxicola*, but dental characters of *Millardia* sp. appear to be more adapted to a grass diet than for the two species of *Mus* (Tables S8 and S9). Shiels [58] reported that *Rattus rattus* has more negative δ^13^C values than coexisting *Mus musculus* although *Rattus* has greater values in VD index than *Mus* (Table S8). Among large herbivorous mammals, hypsodont ungulate species are not universally specialized for consuming C_4_ plants [6], [62]. Feranec [6], [62] proposed that hypsodonty may broaden the dietary niche of generalists feeding on mixed C_3_/C_4_ diets, which may be the case for murine rodents. Further studies of isotopic dietary inference are necessary to understand the ecomorphological strategy of small mammals. However, taxonomically fine-scale comparisons of carbon isotope ratios with grazing indices throughout the temporally well-constrained sequence of Siwalik fossil localities has provided a more detailed and calibrated view of rodent ecomorphology and dietary change than previously possible.

## Supporting Information

Figure S1 **Isotope compositions in a sequence of molars from m1 to m3 from three mandibles (#1 to #3) of Recent** ***Rattus*** **sp. (A) δ^13^C data.** (B) δ^18^O data. Specimens were analyzed in the same analytical run except m1 of #1 and #3, which were run four days after the others. All were right molars except m2 of #2.
(PDF)

Figure S2 **SEM images of m1 of laboratory** ***Mus musculus*** **, which are treated by 0.1 M acetic acid.** The m1specimens were provided by the University of Texas, Southwestern Medical Center. Individuals were sacrificed for a research purpose unrelated to this study.
(PDF)

Figure S3 **Scatter plot of δ^13^C data vs. time, ranging from 9.2 to 6.5 Ma, between** ***Karnimata*** **and the “** ***Progonomys*** **clade”.**
(PDF)

Figure S4 **Scatter plot of van Dam's** [**30**] **index vs. time, ranging from 9.2 to 6.5 Ma, between** ***Karnimata*** **and the “** ***Progonomys*** **clade”.**
(PDF)

Figure S5 **Scatter plot of hypsodonty vs. time, ranging from 9.2 to 6.5 Ma, between** ***Karnimata*** **and the “** ***Progonomys*** **clade”.**
(PDF)

Dataset S1 **Full data of m1 specimens analyzed in this study, including dental measurements, δ^13^C values, and the side of teeth from which isotope data were taken.** The δ^13^C values are corrected to be consistent with the H_3_PO_4_ method using the laser-H_3_PO_4_ offset of modern beaver incisor in every run. Specimens from YGSP 182 treated by 0.1 M acetic acid instead of 0.1 M sodium acetate-acetic acid buffer are marked in Acetic acid.
(TXT)

Dataset S2 **Full data of M1 specimens of Siwalik murines from the Potwar Plateau, including dental measurements, van Dam's** [**30**] **index, and hypsodonty values.** Abbreviations: lap, distance between the posterior side of the lingual anterocone and that of protocone (see Figure 3C); VD, van Dam's [30] index; Hy, hypsodonty.
(TXT)

Table S1 **Key to names of Siwalik murine species used in this study in comparison to Jacobs and Flynn** [**29**] **.** Major species are those known by five or more specimens of the upper first molars (M1). Minor species are those known by less than five specimens of M1.
(PDF)

Table S2 **Carbon isotope data summarized by age.** Note that all data but YGSP 34415 are included in the descriptive statistics.
(PDF)

Table S3 **Results of ANCOVA between** ***Karnimata*** **and the “** ***Progonomys*** **clade”.** Asterisks for *p*<0.05.
(PDF)

Table S4 **Summary of linear regression analysis for δ^13^C values, VD index, and hypsodonty.** Asterisks for *p*<0.05.
(PDF)

Table S5 **Results of statistical tests for carbon isotope data in coexisting species at 7.4 Ma, 6.5 Ma, and Recent.** Asterisks for *p*<0.05. Abbreviations: WA, Welch's ANOVA; MW, Mann-Whitney U test.
(PDF)

Table S6 **Results of the Games-Howell post-hoc test, associated with Table S5.** The mean difference is expressed as a mean of species 1 minus that of species 2. Asterisks for *p*<0.05.
(PDF)

Table S7 **Summary of dental measurements of m1 in mm.**
(PDF)

Table S8 **Means of VD index with 95 % bootstrap confidence intervals.**
(PDF)

Table S9 **Means of hypsodonty measurements with 95 % bootstrap confidence intervals.** Asterisks indicate hypsodonty values measured in specimens in wear stage III of Lazzari et al. [35].
(PDF)

Supporting Information S1 **Effect of nursing on carbon isotope composition in murine rodents.**
(PDF)
