# Supplementary material for: Fossil Mice and Rats Show Isotopic Evidence of Niche Partitioning and Change in Dental Ecomorphology Related to Dietary Shift in Late Miocene of Pakistan
Source: PLoS One. 2013 Aug 2;8(8):e69308. doi: 10.1371/journal.pone.0069308 (PMC3732283; doi:10.1371/journal.pone.0069308)
Supplement: Figure S1 — Isotope compositions in a sequence of molars from m1 to m3 from three mandibles (#1 to #3) of Recent Rattus sp. (A) δ13C data. (B) δ18O data. Specimens were analyzed in the same analytical run except m1 of #1 and #3, which were run four days after the others. All were right molars except m2 of #2. (PDF) [file pone.0069308.s001.pdf]

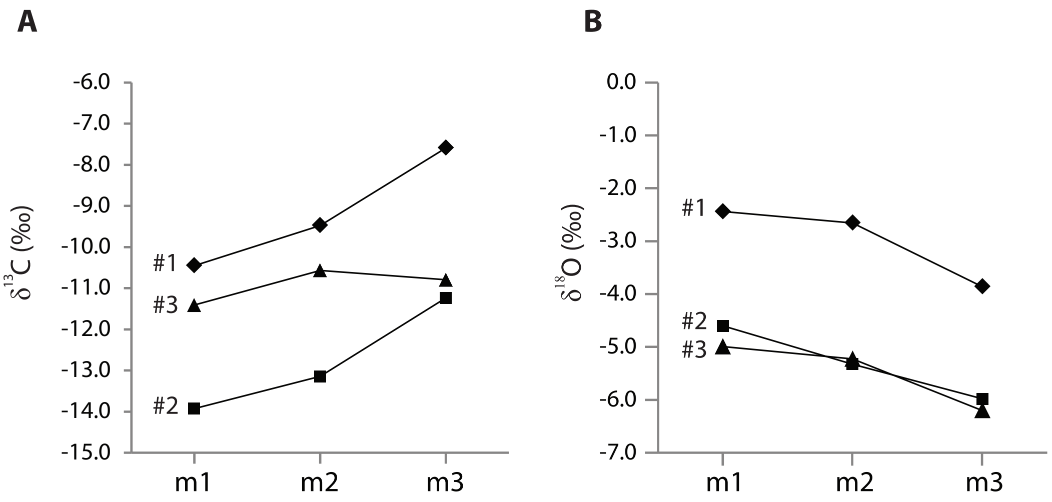

Figure S1. Isotope compositions in a sequence of molars from m1 to m3 from three mandibles (#1 to #3) of Recent *Rattus* sp. (A) $\delta^{13}C$ data. (B) $\delta^{18}O$ data. Specimens were analyzed in the same analytical run except m1 of #1 and #3, which were run four days after the others. All were right molars except m2 of #2.
