# Supplementary material for: Fossil Mice and Rats Show Isotopic Evidence of Niche Partitioning and Change in Dental Ecomorphology Related to Dietary Shift in Late Miocene of Pakistan
Source: PLoS One. 2013 Aug 2;8(8):e69308. doi: 10.1371/journal.pone.0069308 (PMC3732283; doi:10.1371/journal.pone.0069308)
Supplement: Figure S2 — SEM images of m1 of laboratory Mus musculus , which are treated by 0.1 M acetic acid. The m1specimens were provided by the University of Texas, Southwestern Medical Center. Individuals were sacrificed for a research purpose unrelated to this study. (PDF) [file pone.0069308.s002.pdf]

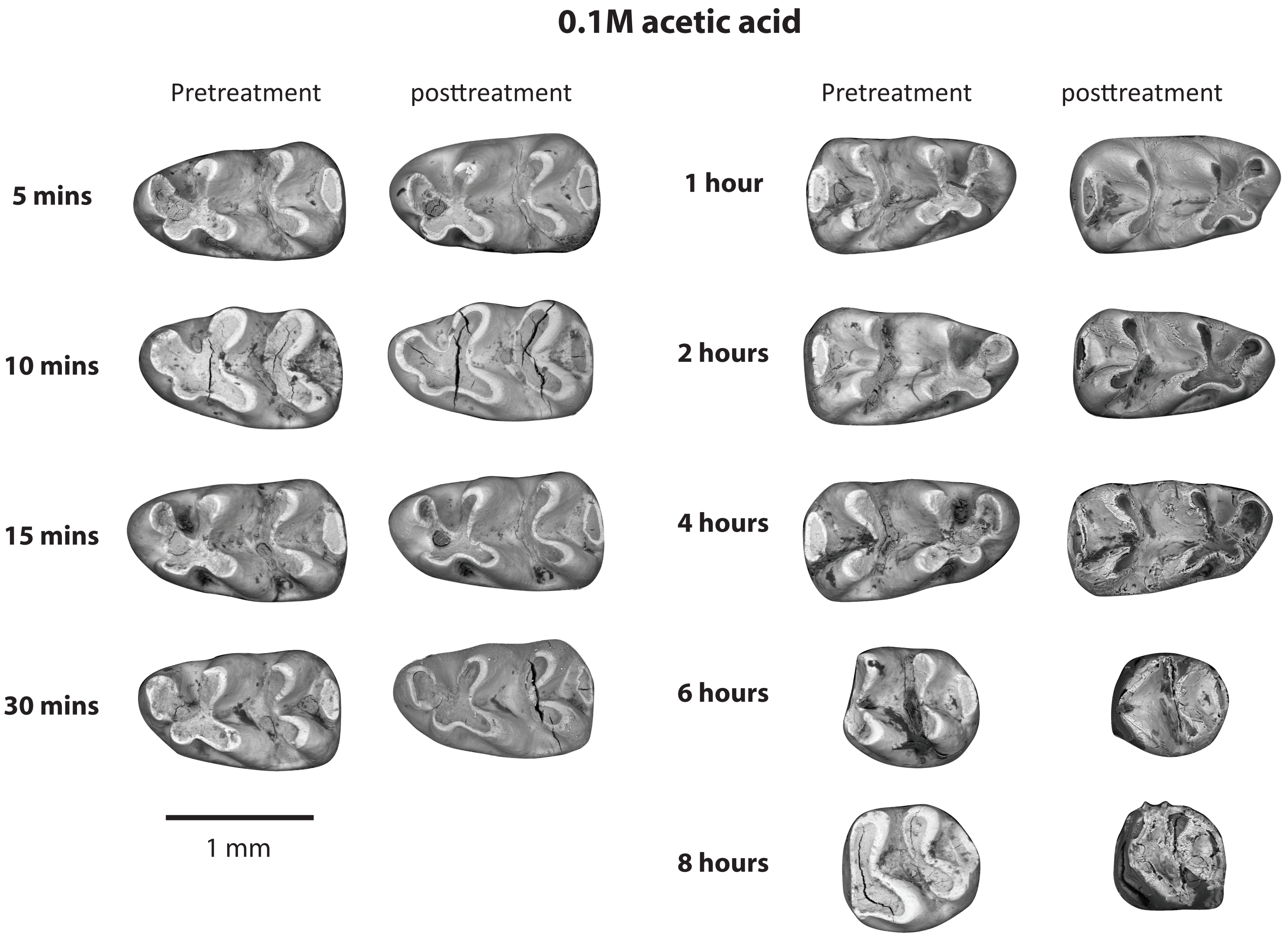

Figure S2. SEM images of m1 of laboratory *Mus musculus*, which are treated by 0.1 M acetic acid. The m1specimens were provided by the University of Texas, Southwestern Medical Center. Individuals were sacrificed for a research purpose unrelated to this study.
