# Supplementary material for: Fossil Mice and Rats Show Isotopic Evidence of Niche Partitioning and Change in Dental Ecomorphology Related to Dietary Shift in Late Miocene of Pakistan
Source: PLoS One. 2013 Aug 2;8(8):e69308. doi: 10.1371/journal.pone.0069308 (PMC3732283; doi:10.1371/journal.pone.0069308)
Supplement: Figure S3 — Scatter plot of δ13C data vs. time, ranging from 9.2 to 6.5 Ma, between Karnimata and the “ Progonomys clade”. (PDF) [file pone.0069308.s003.pdf]

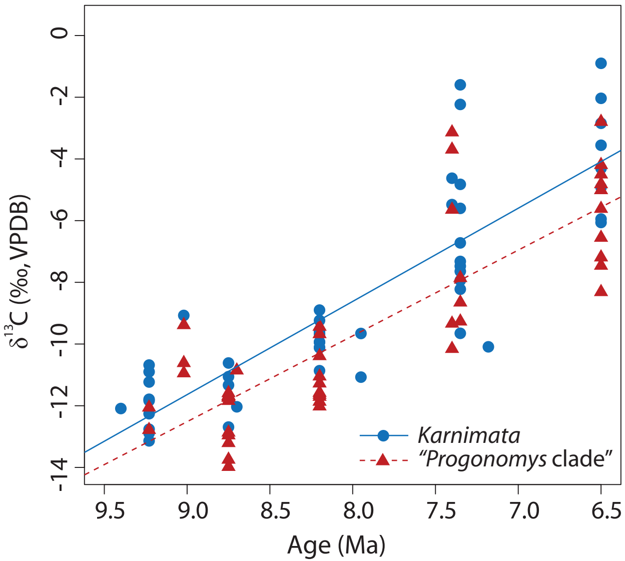

Figure S3. Scatter plot of $\delta^{13}C$ data vs. time, ranging from 9.2 to 6.5 Ma, between *Karnimata* and the "*Progonomys* clade". Lines show linear regression lines.
