# Supplementary material for: Fossil Mice and Rats Show Isotopic Evidence of Niche Partitioning and Change in Dental Ecomorphology Related to Dietary Shift in Late Miocene of Pakistan
Source: PLoS One. 2013 Aug 2;8(8):e69308. doi: 10.1371/journal.pone.0069308 (PMC3732283; doi:10.1371/journal.pone.0069308)
Supplement: Figure S4 — Scatter plot of van Dam's [30] index vs. time, ranging from 9.2 to 6.5 Ma, between Karnimata and the “ Progonomys clade”. (PDF) [file pone.0069308.s004.pdf]

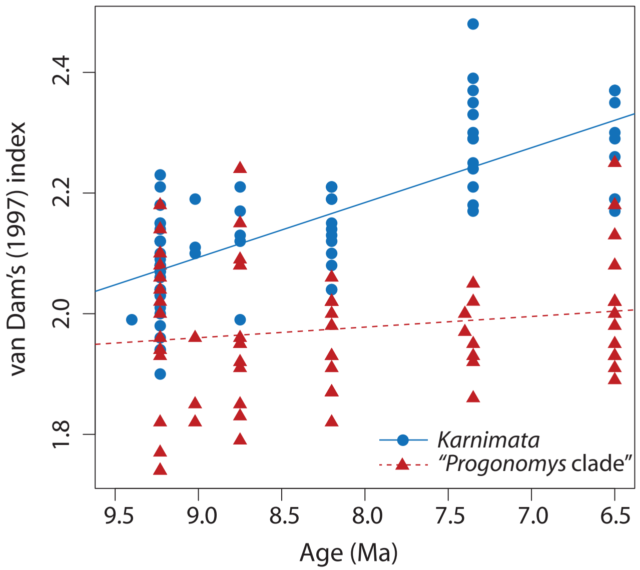

Figure S4. Scatter plot of van Dam's (1997) index vs. time, ranging from 9.2 to 6.5 Ma, between *Karnimata* and the "*Progonomys* clade". Lines show linear regression lines.
