# Supplementary material for: Fossil Mice and Rats Show Isotopic Evidence of Niche Partitioning and Change in Dental Ecomorphology Related to Dietary Shift in Late Miocene of Pakistan
Source: PLoS One. 2013 Aug 2;8(8):e69308. doi: 10.1371/journal.pone.0069308 (PMC3732283; doi:10.1371/journal.pone.0069308)
Supplement: Table S1 — Key to names of Siwalik murine species used in this study in comparison to Jacobs and Flynn [29] . Major species are those known by five or more specimens of the upper first molars (M1). Minor species are those known by less than five specimens of M1. (PDF) [file pone.0069308.s008.pdf]

Table S1. Key to names of Siwalik murine species used in this study in comparison to Jacobs and Flynn (2005). Major species are those known by five or more specimens of the upper first molars (M1). Minor species are those known by less than five specimens of M1.

Locality	Age (Ma)	Jacobs and Flynn (2005)	Major species (n>5 by M1)	Minor species (n<5 by M1)
DP13	6.5	*Pararobertsi robertsi*	*Pararobertsi robertsi*	
		Karnimata huxleyi	*Karnimata huxleyi*	
		Mus auctor	*Mus auctor*	
YGSP 581	7.2		*Karnimata* sp.	
			Mus sp.	
YGSP 931, 921	7.4	*Parapelomys* cf. *P. robertsi*		*Parapelomys* sp.
		Karnimata sp.	*Karnimata* sp.	
			Progonomys sp.	
		Mus sp.	*Mus* sp.	
YGSP 457	7.4	*Parapelomys* cf. *P. robertsi*		*Parapelomys* sp.
		Karnimata sp.	*Karnimata* sp.	
			Progonomys sp.	
		Mus sp.	*Mus* sp.	
				morphotype 4
YGSP 898	7.8			morphotype 8
YGSP 547	8.0			*Parapelomys* sp.
				Karnimata sp.
				morphotype 8
				Mus sp.
YGSP 24	8.2	*Parapelomys* sp.		large *Karnimata* sp.
		Karnimata sp.	*Karnimata* sp.	
		Progonomys sp.	*Progonomys* sp.	
YGSP 387	8.7			*Karnimata* sp.
				morphotype 7
				Progonomys sp.
YGSP 388	8.8	large *Karnimata* sp.		large *Karnimata* sp.
				morphotype 4
				morphotype 7
		Karnimata sp.	*Karnimata* sp.	
		Progonomys sp.	*Progonomys* sp.	
YGSP 367	9.0	large *Karnimata* sp.		
		Karnimata darwini	*Karnimata darwini*	
		Progonomys debruijni	*Progonomys debruijni*	
YGSP 182	9.2	*Parapodemus* sp.		*Parapodemus* sp.
		large *Karnimata* sp.		large *Karnimata* sp.
		Karnimata darwini	*Karnimata darwini*	
		Progonomys debruijni	*Progonomys debruijni*	
YGSP 410	9.4		*Karnimata darwini*	
			Progonomys debruijni	
YGSP 311	10.1	*Karnimata* sp.	*Karnimata* sp.	
		Progonomys sp.	*Progonomys* sp.	
		unnamed sp.		morphotype 2
YGSP 450	10.2	*Karnimata* sp.	*Karnimata* sp.	
		Progonomys sp.	*Progonomys* sp.	
YGSP 259	10.5	*Karnimata* sp.	*Karnimata* sp.	
		Progonomys sp.	*Progonomys* sp.	
				morphotype 1

YGSP 797	11.2	? *Karnimata* sp.	? *Karnimata* sp.	
		Progonomys hussaini	*Progonomys hussaini*	
YGSP 791	11.2		same as Y76	
YGSP 809	11.3		same as Y76	
YGSP 76	11.4	*Progonomys hussaini*	*Progonomys hussaini*	
YGSP 634	12.3	*Progonomys* sp.		*Progonomys* sp.
YGSP 496	12.4	near *Progonomys*	near *Progonomys*	
YGSP 825	12.8		*Antemus chinjiensis*	
YGSP 690	13.1	*Antemus chinjiensis*	*Antemus chinjiensis*	
YGSP 718	13.2	*Antemus chinjiensis*	*Antemus chinjiensis*	
YGSP 651	13.6	*Antemus chinjiensis*	*Antemus chinjiensis*	
YGSP 430, 41	13.6	*Antemus chinjiensis*	*Antemus chinjiensis*	
YGSP 59, 640, 641	13.7	*Antemus chinjiensis*	*Antemus chinjiensis*	
YGSP 491	13.8	*Antemus chinjiensis*	*Antemus chinjiensis*	
