# Supplementary material for: Fossil Mice and Rats Show Isotopic Evidence of Niche Partitioning and Change in Dental Ecomorphology Related to Dietary Shift in Late Miocene of Pakistan
Source: PLoS One. 2013 Aug 2;8(8):e69308. doi: 10.1371/journal.pone.0069308 (PMC3732283; doi:10.1371/journal.pone.0069308)
Supplement: Table S2 — Carbon isotope data summarized by age. Note that all data but YGSP 34415 are included in the descriptive statistics. (PDF) [file pone.0069308.s009.pdf]

Table S2. Carbon isotope data summarized by age. Note that all data but YGSP 34415 are included in the descriptive statistics.

Age (Ma)	N	$\delta^{13}C$			Combined locality/age
		Mean	SD	Range	
Recent Pakistan	16	-5.8	5.1	14.3	
Recent India	2	-2.1	0.0	0.0	
7.4 to 6.5	50	-6.3	2.6	9.8	
13.8 to 7.8	110	-10.8	1.3	6.0	
~2.5 to ~1.8	11	-4.3	1.7	5.8	loc. Kanthro, loc. Nadah
6.5	22	-5.4	2.2	8.1	
7.4	25	-6.6	2.5	9.0	
8.0	5	-10.4	1.1	2.6	
8.2	19	-10.5	1.0	3.1	YGSP 34415 removed as an outlier
8.8	18	-12.0	1.0	3.5	8.7 Ma, 8.8 Ma
9.0	4	-10.0	0.9	1.9	
9.2	11	-12.0	0.8	2.5	
10.1	10	-9.6	0.5	1.5	
10.2	3	-10.4	0.2	0.4	
10.5	10	-11.0	0.9	3.3	
11.2	8	-10.4	0.8	2.4	
11.4	10	-10.6	0.8	2.6	11.3 Ma, 11.4 Ma
13.8	8	-9.5	0.9	3.3	
