# Supplementary material for: Fossil Mice and Rats Show Isotopic Evidence of Niche Partitioning and Change in Dental Ecomorphology Related to Dietary Shift in Late Miocene of Pakistan
Source: PLoS One. 2013 Aug 2;8(8):e69308. doi: 10.1371/journal.pone.0069308 (PMC3732283; doi:10.1371/journal.pone.0069308)
Supplement: Table S3 — Results of ANCOVA between Karnimata and the “ Progonomys clade”. Asterisks for p<0.05. (PDF) [file pone.0069308.s010.pdf]

Table S3. Results of ANCOVA between *Karnimata* and the "*Progonomys* clade". Asterisks for $p<0.05$.

Dependent variable	Interaction		ANCOVA
	Covariate:Categorical variable	*p*	*p*
delta ^{13}C	Age:Clade	0.54	0.002*
VD index	Age:Clade	<0.001*	
Hypsodonty	Age:Clade	0.39	0.04*
