# Supplementary material for: Fossil Mice and Rats Show Isotopic Evidence of Niche Partitioning and Change in Dental Ecomorphology Related to Dietary Shift in Late Miocene of Pakistan
Source: PLoS One. 2013 Aug 2;8(8):e69308. doi: 10.1371/journal.pone.0069308 (PMC3732283; doi:10.1371/journal.pone.0069308)
Supplement: Table S4 — Summary of linear regression analysis for δ13C values, VD index, and hypsodonty. Asterisks for p<0.05. (PDF) [file pone.0069308.s011.pdf]

Table S4. Summary of linear regression analysis for $\delta^{13}C$ values, VD index, and hypsodonty. Asterisks for $p<0.05$.

Clade	Dependent variable	Independent variable	Slope	Intercept	R^2	*p*
Karnimata	delta ^{13}C	Age	-3.02	15.5	0.73	<0.001*
"*Progonomys* clade"	delta ^{13}C	Age	-2.78	12.5	0.69	<0.001*
Karnimata	VD index	Age	-0.09	2.91	0.56	<0.001*
"*Progonomys* clade"	VD index	Age	-0.02	2.12	0.02	0.22
Karnimata	Hypsodonty	Age	0.003	0.37	<0.01	0.53
"*Progonomys* clade"	Hypsodonty	Age	0.008	0.31	0.08	0.06
