# Supplementary material for: Fossil Mice and Rats Show Isotopic Evidence of Niche Partitioning and Change in Dental Ecomorphology Related to Dietary Shift in Late Miocene of Pakistan
Source: PLoS One. 2013 Aug 2;8(8):e69308. doi: 10.1371/journal.pone.0069308 (PMC3732283; doi:10.1371/journal.pone.0069308)
Supplement: Table S5 — Results of statistical tests for carbon isotope data in coexisting species at 7.4 Ma, 6.5 Ma, and Recent. Asterisks for p<0.05. Abbreviations: WA, Welch's ANOVA; MW, Mann-Whitney U test. (PDF) [file pone.0069308.s012.pdf]

Table S5. Results of Welch's ANOVA for carbon isotope data in coexisting species at 7.4 Ma, 6.5 Ma, and Recent. Asterisks for *p*<0.05.

Age (Ma)	Species 1		Species 2		Species 3		*p*	Combined age
	Name	n	Name	n	Name	n		
Recent	*Golunda ellioti*	4	*Rattus* sp. + *Millardia* sp.	5	*Mus* spp.	5	<0.001*	
6.5	*Parapelomys robertsi*	4	*Karnimata huxleyi*	8	*Mus auctor*	10	0.001*	
7.4	*Karnimata* sp.	14	*Progonomys* sp.	8	*Mus* sp.	4	0.003*	7.2 Ma, 7.4 Ma
