# Supplementary material for: Fossil Mice and Rats Show Isotopic Evidence of Niche Partitioning and Change in Dental Ecomorphology Related to Dietary Shift in Late Miocene of Pakistan
Source: PLoS One. 2013 Aug 2;8(8):e69308. doi: 10.1371/journal.pone.0069308 (PMC3732283; doi:10.1371/journal.pone.0069308)
Supplement: Table S6 — Results of the Games-Howell post-hoc test, associated with Table S5. The mean difference is expressed as a mean of species 1 minus that of species 2. Asterisks for p<0.05. (PDF) [file pone.0069308.s013.pdf]

Table S6. Results of the Games-Howell post-hoc test, associated with Table S5. The mean difference is expressed as a mean of species 1 minus that of species 2. Asterisks for p<0.05.

Age (Ma)	Species 1	Species 2	Mean difference	p
Recent	*Golunda ellioti*	*Rattus* sp. + *Millardia* sp.	12.6	<0.001*
	Golunda ellioti	*Mus* spp.	4.5	0.01*
	Rattus sp. + *Millardia* sp.	*Mus* spp.	-8.1	<0.001*
6.5	*Parapelomys robertsi*	*Karnimata huxleyi*	-4.3	0.001*
	Parapelomys robertsi	*Mus auctor*	-2.4	0.02*
	Karnimata huxleyi	*Mus auctor*	1.8	0.11
7.4	*Karnimata* sp.	*Progonomys* sp.	0.8	0.76
	Karnimata sp.	*Mus* sp.	3.5	0.002*
	Progonomys sp.	*Mus* sp.	2.6	0.08
