# Supplementary material for: Fossil Mice and Rats Show Isotopic Evidence of Niche Partitioning and Change in Dental Ecomorphology Related to Dietary Shift in Late Miocene of Pakistan
Source: PLoS One. 2013 Aug 2;8(8):e69308. doi: 10.1371/journal.pone.0069308 (PMC3732283; doi:10.1371/journal.pone.0069308)
Supplement: Table S7 — Summary of dental measurements of m1 in mm. (PDF) [file pone.0069308.s014.pdf]

Table S7. Summary of dental measurements of m1 in mm.

Age (Ma)	Species	N	Length	Width	Ln(length*width)			Combined age
					Mean	SD	Range	
Recent	*Golunda ellioti*	5	2.65	1.77	1.54	0.07	0.17	
	Rattus sp.	2	2.74	1.57	1.46	0.12	0.17	
	Millardia sp.	3	2.31	1.49	1.24	0.01	0.03	
	Mus booduga	4	1.44	0.87	0.21	0.03	0.08	
	Mus saxicola	1	1.71	1.09	0.62			
6.5	*Parapelomys robertsi*	4	2.43	1.61	1.36	0.03	0.08	
	Karnimata huxleyi	8	1.85	1.19	0.78	0.13	0.40	
	Mus auctor	12	1.49	0.94	0.34	0.08	0.22	
7.4	*Parapelomys* sp.	1	2.22	1.50	1.20			7.2 Ma, 7.4 Ma
	Karnimata sp.	17	1.94	1.26	0.89	0.09	0.35	
	Progonomys sp.	11	1.68	1.04	0.56	0.15	0.46	
	Mus sp.	12	1.32	0.80	0.04	0.14	0.40	
8.2	large *Karnimata* sp.	2	2.43	1.45	1.26	0.04	0.06	8.0 Ma, 8.2 Ma
	Karnimata sp.	10	2.16	1.38	1.09	0.08	0.25	
	Progonomys sp.	12	1.52	0.93	0.34	0.11	0.34	
8.8	large *Karnimata* sp.	3	2.33	1.37	1.16	0.08	0.15	8.7 Ma, 8.8 Ma
	Karnimata sp.	2	2.25	1.35	1.11	0.08	0.11	
	Progonomys sp.	13	1.52	0.94	0.36	0.07	0.23	
9.2	*Karnimata darwini*	14	1.95	1.19	0.84	0.11	0.37	
	Progonomys debruijni	7	1.36	0.84	0.13	0.09	0.24	
10.1	*Karnimata* sp. + *Progonomys* sp.	20	1.67	1.01	0.52	0.10	0.32	
10.2	*Karnimata* sp. + *Progonomys* sp.	7	1.60	0.96	0.42	0.12	0.33	
10.5	*Karnimata* sp. + *Progonomys* sp.	18	1.60	0.97	0.43	0.10	0.42	
11.2	*Progonomys hussaini* + ? *Karnimata* sp.	15	1.59	0.98	0.44	0.15	0.44	
11.4	*Progonomys hussaini*	16	1.56	0.98	0.42	0.07	0.27	11.3 Ma, 11.4 Ma
13.8	*Antemus chinjiensis*	10	1.52	0.94	0.36	0.10	0.36	
