# Supplementary material for: Fossil Mice and Rats Show Isotopic Evidence of Niche Partitioning and Change in Dental Ecomorphology Related to Dietary Shift in Late Miocene of Pakistan
Source: PLoS One. 2013 Aug 2;8(8):e69308. doi: 10.1371/journal.pone.0069308 (PMC3732283; doi:10.1371/journal.pone.0069308)
Supplement: Table S8 — Means of VD index with 95 % bootstrap confidence intervals. (PDF) [file pone.0069308.s015.pdf]

Table S8. Means of VD index with 95 % bootstrap confidence intervals.

Age (Ma)	Species	N	Mean	Bootstrap confidence interval		SD	Combined age
				Lower	Upper		
Recent	*Golunda ellioti*	3	2.52			0.06	
	Rattus sp.	3	2.36			0.19	
	Millardia sp.	3	2.59			0.16	
	Mus spp.	5	2.01			0.08	
6.5	*Parapelomys robertsi*	5	2.54	2.50	2.59		
	Karnimata huxleyi	10	2.29	2.24	2.33		
	Mus auctor	13	2.02	1.98	2.08		
7.4	*Karnimata* sp.	13	2.30	2.25	2.35		
	Progonomys sp.	8	1.96	1.93	2.00		
	Mus sp.	9	1.77	1.72	1.86		
8.2	*Karnimata* sp. (+ large *Karnimata* sp.)	14	2.13	2.09	2.18		
	Progonomys sp.	11	1.95	1.91	1.98		
8.8	*Karnimata* sp. (+ large *Karnimata* sp.)	5	2.12	2.04	2.17		
	Progonomys sp.	14	1.99	1.94	2.05		
9.2	*Karnimata darwini*	33	2.07	2.04	2.10		
	Progonomys debruijni	15	1.97	1.89	2.04		
10.1	*Karnimata* sp.	9	2.01	1.95	2.06		
10.5	*Karnimata* sp.	11	1.95	1.91	2.01		10.2 Ma, 10.5 Ma
	Progonomys sp.	12	1.95	1.90	1.99		
11.2	*Progonomys hussaini*	9	2.02	1.99	2.06		
	? *Karnimata* sp.	6	2.15	2.08	2.21		
11.4	*Progonomys hussaini*	21	2.00	1.93	2.07		11.3 Ma, 11.4 Ma
11.6	*Progonomys hussaini*	5	1.97	1.88	2.12		11.5 Ma, 11.6 Ma, 12.3 Ma
12.4	near *Progonomys*	5	2.05	1.97	2.15		
13.0	*Antemus chinjiensis*	7	2.02	1.95	2.07		12.8 Ma, 13.1 Ma, 13.2 Ma
13.6	*Antemus chinjiensis*	14	1.99	1.95	2.03		13.6 Ma, 13.7 Ma
13.8	*Antemus chinjiensis*	12	2.02	1.97	2.07		
