# Supplementary material for: Fossil Mice and Rats Show Isotopic Evidence of Niche Partitioning and Change in Dental Ecomorphology Related to Dietary Shift in Late Miocene of Pakistan
Source: PLoS One. 2013 Aug 2;8(8):e69308. doi: 10.1371/journal.pone.0069308 (PMC3732283; doi:10.1371/journal.pone.0069308)
Supplement: Table S9 — Means of hypsodonty measurements with 95 % bootstrap confidence intervals. Asterisks indicate hypsodonty values measured in specimens in wear stage III of Lazzari et al. [35]. (PDF) [file pone.0069308.s016.pdf]

Table S9. Means of hypsodonty measurements with 95 % bootstrap confidence intervals. Asterisks indicate hypsodonty values measured in specimens in wear stage III of Lazzari et al. (2008).

Age (Ma)	Species	N	Mean	Bootstrap confidence interval		Combined age
				Lower	Upper	
Recent	*Goluda ellioti*	1	0.42*			
	Millardia sp.	1	0.45			
	Mus booduga	1	0.37*			
6.5	*Parapelomys robertsi*	4	0.46	0.42	0.47	
	Karnimata huxleyi	6	0.39	0.38	0.43	
	Mus auctor	5	0.36	0.35	0.38	
7.4	*Karnimata* sp.	8	0.39	0.38	0.42	
	Progonomys sp.	5	0.37	0.35	0.39	
	Mus sp.	5	0.36	0.35	0.37	
8.2	*Karnimata* sp. (+ large *Karnimata* sp.)	8	0.38	0.37	0.40	
	Progonomys sp.	7	0.38	0.37	0.39	
8.8	*Progonomys* sp.	5	0.39	0.38	0.40	
9.2	*Karnimata darwini*	22	0.39	0.38	0.40	
	Progonomys debruijni	8	0.39	0.37	0.40	
10.5	*Progonomys* sp.	9	0.38	0.37	0.39	
11.2	*Progonomys hussaini*	7	0.40	0.38	0.41	
11.4	*Progonomys hussaini*	7	0.37	0.36	0.39	11.3 Ma, 11.4 Ma
13.0	*Antemus chinjiensis*	5	0.38	0.36	0.40	12.8 Ma, 13.1 Ma, 13.2 Ma
13.6	*Antemus chinjiensis*	9	0.36	0.34	0.38	13.6 Ma, 13.7 Ma
