# Supplementary material for: Fossil Mice and Rats Show Isotopic Evidence of Niche Partitioning and Change in Dental Ecomorphology Related to Dietary Shift in Late Miocene of Pakistan
Source: PLoS One. 2013 Aug 2;8(8):e69308. doi: 10.1371/journal.pone.0069308 (PMC3732283; doi:10.1371/journal.pone.0069308)
Supplement: Supporting Information S1 — Effect of nursing on carbon isotope composition in murine rodents. (PDF) [file pone.0069308.s017.pdf]

Fossil Mice and Rats Show Isotopic Evidence of Niche Partitioning and Change in Dental Ecomorphology Related to Dietary Shift in late Miocene of Pakistan

Kimura et al.

Supporting Information S1

Effect of Nursing on Carbon Isotope Composition in Murine Rodents

Developmental timing of molars greatly overlaps with lactation in murine rodents, in which deciduous cheek teeth are lacking. In mice, enamel mineralization of m1 and m2 begins only a few days after birth and completes within 16 to 18 days while mother's milk was their only food source, and m3 begins enamel mineralization in Day 11 to 12 and erupts in Day 28 to 29 [1]. Thus, isotope compositions in m1 and m2 would reflect nutrients from mother's milk, and $\delta^{13}C$ values of m3 would partially reflect solid diet after weaning. Milk is depleted in ^{13}C by 2.1 ‰ relative to the mother's plasma [2] because milk has a high lipid content, which is depleted relative to bulk diet [3,4]. Teeth formed before weaning have lower $\delta^{13}C$ values than those formed after weaning by ~0.5 ‰ in humans [5] to ~2 ‰ in sea lions [6]. The varying degree of the milk effect probably comes from varying fat contents of milk [7]. In fossil theridomyid rodents, the deciduous premolar is depleted in ^{13}C by 1.5 ‰ relative to the permanent molar [8].

We measured isotope ratios in lower molars (m1, m2, m3) of three specimens of Recent *Rattus* sp. from Pakistan, whose diets are unknown (Figure S1). The result that $\delta^{13}C$ values in m1 were more negative than $\delta^{13}C$ in m3 (Figure S1A) is consistent with the timing of enamel mineralization. The isotope enrichment between m1 and m3 ($^{13}\delta^{*}_{m1\text{-}m3}$) varies from -0.6 ‰ (n=1) to -2.7 and -2.9 ‰ (n=2), which encompasses the total variation in teeth formed before and after weaning of different animals. The isotope enrichment of m3-m2 (1.9 ‰, n=2) is larger than that of m2-m1 (0.9 ‰), suggesting that carbon isotope ratios in m3 reflect, if not fully, post-weaning diet. A slight enrichment of m2-m1 may come from an analytical uncertainty or a partial signal of prenatal nutrients transported through mother's placenta. Thus, it suggests that carbon isotope compositions in m1of murine rodents are influenced by mother's milk.

The result that the mean $\delta^{18}O$ value of m1 is more positive than that of m3 by 1.3 ‰ (Figure S1B) is also congruent with the fact that isotope composition in m1 is more influenced by mother's milk. Milk is synthesized from mothers' body water, whose isotope composition is more enriched in ^{18}O than that of drinking water due to water intake from plants and metabolic reactions in the body [9,10]. Based on a model modified from Bryant and Froelich [10] to describe the influence of seasonal birth on oxygen isotope composition in different dentitions, Bryant et al. [11] demonstrated that, if individuals are born in the same season, oxygen composition in teeth formed prior to weaning are more enriched in ^{18}O than teeth formed after weaning.

Carbon isotope values in large herbivorous mammals are often used to estimate the proportion of C_3 plants (all trees, most shrubs, and some grasses) versus C_4 grasses in their diet based on a two-source linear mixing model [12,13,14,15] with isotope enrichment between diet and bioapatite [16]. Our brief analysis on a sequence of lower molars of Recent *Rattus* sp. suggests that

the isotopic effect of nursing on molars must be understood well to reconstruct the proportion of C_3 to C_4 diet of small mammals using molars rather than ever-growing incisors.

References

1. Cohn SA (1957) Development of the molar teeth in the albino mouse. Am J Anat 101: 295-319.
2. Jenkins SG, Partridge ST, Stephenson TR, Farley SD, Robbins CT (2001) Nitrogen and carbon isotope fractionation between mothers, neonates, and nursing offspring. Oecologia 129: 336-341.
3. DeNiro MJ, Epstein S (1977) Mechanism of carbon isotope fractionation associated with lipid synthesis. Science 197: 261-263.
4. Tieszen LL, Fagre T (1993) Effects of diet quality and composition on the isotopic composition of respiratory CO_2, bone collagen, bioapatite and soft tissue. In: Lambert JB, Grupe G, editors. Prehistoric Human Bone: Archaeology at the Molecular Level. Berlin: Springer-Verlag. pp. 121-155.
5. Wright LE, Schwarcz HP (1998) Stable carbon and oxygen isotopes in human tooth enamel: identifying breastfeeding and weaning in prehistory. Am J Phys Anthropol 106: 1-18.
6. Hobson KA, Sease JL (1998) Stable carbon isotope analyses of tooth annuli reveal temporal dietary records: an example using Steller sea lions. Mar Mammal Sci 114: 116-129.
7. Balasse M (2002) Reconstructing dietary and environmental history from enamel isotopic analysis: Time resolution of intra-tooth sequential sampling. Int J Osteoarchaeol 12: 155-165.
8. Grimes ST, Collinson ME, Hooker JJ, Mattey DP, Grassineau NV, et al. (2004) Distinguishing the diets of coexisting fossil theridomyid and glirid rodents using carbon isotopes. Palaeogeogr Palaeoclimatol Palaeoecol 208: 103-119.
9. D'Angela D, Longinelli A (1990) Oxygen isotopes in living mammal's bone phosphate: Further results. Chem Geol 86: 75-82.
10. Bryant JD, Froelich PN (1995) A model of oxygen isotope fractionation in body water of large mammals. Geochim Cosmochim Acta 59: 4523-4537.
11. Bryant JD, Froelich PN, Showers WJ, Genna BJ (1996) Biologic and climatic signals in the oxygen isotopic composition of Eocene-Oligocene equid enamel phosphate. Palaeogeogr Palaeoclimatol Palaeoecol 126: 75-89.
12. Passey BH, Cerling TE, Perkins ME, Voorhies MR, Harris JM, et al. (2002) Environmental change in the Great Plains: An isotopic record from fossil horses. J Geol 110: 123-140.
13. Cerling TE, Harris JM, Leakey MG, Mudida N (2003) Stable isotope ecology of Northern Kenya, with emphasis on the Turkana Basin. In: Leakey MG, Harris JM, editors. Lothagam: The Dawn of Humanity in East Africa: Columbia University Press. pp. 583-603.
14. Feranec RS (2003) Stable isotopes, hypsodonty, and the paleodiet of *Hemiauchenia* (Mammalia: Camelidae): a morphological specialization creating ecological generalization. Paleobiology 29: 230-242.
15. Hynek SA, Passey BH, Prado JL, Brown FH, Cerling TE, et al. (2012) Small mammal carbon isotope ecology across the Miocene–Pliocene boundary, northwestern Argentina. Earth Planet Sci Lett 321-322: 177-188.
16. Cerling TE, Harris JM (1999) Carbon isotope fractionation between diet and bioapatite in ungulate mammals and implications for ecological and paleoecological studies. Oecologia 120: 347-363.
